# Immune system activation by natural products and complex fractions: a network pharmacology approach in cancer treatment

**DOI:** 10.15698/cst2020.07.224

**Published:** 2020-05-18

**Authors:** Alejandra Gomez-Cadena, Alfonso Barreto, Susana Fiorentino, Camilla Jandus

**Affiliations:** 1 Department of Pathology and Immunology, Targeting of Cytokine Secreting Lymphocyte group, Geneva University, Geneva, Switzerland.; 2 Ludwig Institute for Cancer Research, Lausanne Branch, University of Lausanne, Switzerland.; 3 Departamento de Microbiología, Grupo de Inmunobiología y Biología Celular, Pontificia Universidad Javeriana, Bogotá, Colombia.

**Keywords:** natural products, cancer, immune response, network pharmacology, immunomodulation, herbal medicine, immunogenic cell death

## Abstract

Natural products and traditional herbal medicine are an important source of alternative bioactive compounds but very few plant-based preparations have been scientifically evaluated and validated for their potential as medical treatments. However, a promising field in the current therapies based on plant-derived compounds is the study of their immunomodulation properties and their capacity to activate the immune system to fight against multifactorial diseases like cancer. In this review we discuss how network pharmacology could help to characterize and validate natural single molecules or more complex preparations as promising cancer therapies based on their multitarget capacities.

## INTRODUCTION

Over the past decade, immunotherapy has become a promising approach to treat cancer and is now being combined with standard chemotherapy and immune response activators to improve the outcome for cancer patients [1]. However, the rate of drug candidates that are successfully translated into clinics advance slowly due to the lack of efficacy and the clinical safety or toxicology. Another factor that contributes to this phenomenon is the assumption that the main aim for new treatments is to find a quite selective ligand or immune checkpoint that blocks a single target or that stimulates a single pathway [2, 3]. This philosophy of “one gene, one drug, one disease” arises from combining genetic reductionism and molecular biology technologies that allow us to isolate and characterize “disease-causing” genes and is justified as the safest approach to minimize off-target effects. However, until now no “magic bullet” has been described [2].

Although produced by the plant itself to adapt and interact with the environment, many natural compounds have been used as drugs by humans. In the area of cancer treatment, it has been shown that around 75% of the medications available are derived or inspired from plants [4]. Today, one of the main advantages of natural products relies on their multitarget activity allowing the interaction of one compound or a complex fraction with multiple proteins and receptors [5].

Over time, herbal traditional medicine has been considered an important alternative to classical western medicine. Although some herbal preparations are very popular in our society, very few plant-based preparations have been scientifically evaluated and validated for their potential as medical treatments. In most countries herbal drugs are poorly regulated and their safety remains a major concern [6]. However, a promising field in the current therapies based on plant-derived compounds is the study of their synergistic activity when administered in combination with synthetic anticancer medications [7].

In this review, we discuss the importance of the immune system activation by natural compounds in cancer treatment in the context of network pharmacology. We focus on the multitargeting concept and synergistic activity as key tools to treat multifactorial and complex diseases such as cancer.

## THE IMMUNE SYSTEM AND CANCER

The interactions between immunity and cancer have been the object of numerous studies in pre-clinical models to understand how the immune system can repress tumor development and exert its protective function. However, in recent years, it also became clear that the immune system can shape and promote cancer development [8].

In 1909 Paul Ehrlich formulated the initial hypothesis that host defense may prevent neoplastic cells from developing into tumors [9]. This idea was refined by the theory of immune surveillance against cancer proposed by Burnet in 1970 [10], followed by the concept of “3E cancer immunoediting” introduced by Dunn and Schreiber in 2002 [11]. This last theory postulated that the immune system is able to keep in check the development of transformed cells and prevent neoplasia during the initial phase of elimination. Then, due to genetic instability, cells that are more resistant to the immune attack emerge but remain under the constant control of the immune system, during an equilibrium phase. The long-term exposure to this “editor” environment, favors new populations of tumor variants that occasionally will escape the immune system by different mechanisms and become clinically detectable and disseminate [11].

Since then, important experiments demonstrated that endogenous levels of IFN-γ were sufficient to provide protection against the development of transplanted tumors in immunocompetent animals [12]. Further, studies in RAG-2^-/-^ mice showed the importance of T lymphocytes in tumor immune surveillance [13]. Nowadays, the role of the immune system in cancer development has been largely acknowledged and clinically exploited for cancer patient's treatment. Even if we are still a long way from understanding all the underlying mechanisms of antitumor immunity, the current knowledge allows the development of strategies to boost antitumor immune responses in different ways as we will discuss in this review.

## IMMUNE SYSTEM ACTIVATION BY IMMUNOGENIC CELL DEATH (ICD)

Deregulated cell death is a common feature of many human diseases and it is associated with hyper-proliferative disorders, such as cancer [14]. One of the underlying mechanisms proposed by Hanahan and colleagues is the unresponsiveness of tumor cells to the control pathways that regulate proliferation and cell death [15]. Cell death can be classified according to its morphological appearance, enzymatic criteria, functional aspects or immunological characteristics. Several types of cell deaths have been reported but there is an increase interest in understanding which are the cancer cells that are susceptible to death, where, how, and which immune cells are able to “recognize” them and present their tumor-associated antigens to ultimately activate the immune response [16].

Two major barriers usually avoid the malignant transformation of cells in the human body. First, the activation of oncogenes often stimulates a DNA damage response that finally culminates in apoptosis and hence elimination of the mutated cells. Second, the immune system recognizes transformed cells, based on the tumor-specific expression of abnormal or mutated antigens and damage associated molecular patterns (DAMPs) which can trigger the immune response [17]. Little more than a decade ago it was shown that chemotherapy induces a type of cell death, called immunogenic cell death (ICD) that, at least in some cases, activates effector mechanisms directed against dying tumor cells, required for an optimal therapeutic effect [18].

The concept of ICD capable of converting tumors into an *in situ* vaccine was proposed as an efficient way to induce the activation of endogenous immune responses. This type of cell death is characterized by the induction of different DAMPs mediated by endoplasmic reticulum (ER) stress and autophagy, which stimulates cross presentation of tumor cell-derived antigens by dendritic cells (DCs) [19]. ICD is in fact a multifactorial process that involves changes in the composition of the cell membrane and the secretion of soluble factors in a well-defined timeline.

First, the translocation of calreticulin (CRT) from the perinuclear ER to the cancer cell membrane concomitantly with the re-localization of ERp57 will work as an “eat me” signal to induce phagocytosis by DCs, along with the induction of antigen presentation to activate antigen-specific T cells [20]. During the blebbing phase of cell death, autophagy-dependent ATP secretion will enter the equation to play the role of the “find me” signal. During the autophagy process the complex autophagolysosome will fuse with the plasma membrane allowing the release of the “find me” signal [21]. ATP will act as chemoattractant and activate the purinergic receptor P2X7 on DCs triggering NALP3-ASC (nacht domain-, leucine-rich repeat-, and pyrin domain containing protein 3; speck-like protein) inflammasome activation and the production of IL-1β [22]. This pathway will provide cytokines that are necessary in the context of antigen presentation [23]. Moreover, during the late stages of apoptosis, High-mobility group box 1 protein (HMGB1) can be released from the nucleus to the cytoplasm and finally to the extracellular environment to bind to the Toll Like Receptor 4 (TLR4) expressed on DCs to induce their activation. This binding will trigger MyD88 signaling to enhance antigen processing and antigen presentation by inhibiting the fusion of the phagosomes and lysosomes facilitating the cargo of antigens for presentation [24, 25]. The expression of ecto-HSP70 and -HSP90 on the tumor cell membrane also contributes to the tumor clearance through the induction of a tumor-specific CD8 T cell response [26].

### Classical ICD inducers

The traditional view of anticancer therapy as an immunosuppressive or tolerogenic regimen has been challenged by several lines of evidence indicating that some cytotoxic drugs trigger an effective immune response by inducing apoptotic tumor cell death [27]. Today, it is well known that in response to many chemotherapeutic and cell damaging agents like etoposide, mitomycin C, thapsigargin or cisplatin, tumor cells die in a non-immunogenic fashion. However, cancer cells treated with anthracyclines such as doxorubicin, mitoxantrone, oxaliplatin or ionizing irradiation can induce a strong antitumor immune response. Kroemer and collaborators showed in a series of experiments that anthracycline-treated cells injected subcutaneously in immunocompetent mice, in the absence of any adjuvant, are able to induce immune system activation, acting as an “*in situ* vaccine” [26, 27, 28].

To date, several screening studies have been carried out to discover *bona fide* ICD inducers and revealed the inherent capacity of a wide variety of drugs to induce cell death-associated exposure of danger signals and to drive *in vivo* anticancer immune responses [28]. Two kinds of ICD inducers have been described, type I and type II. Anthracyclines and analogues are classified as ICD inducers type I, while the photodynamic therapy (PDT) or the oncolytic viruses are type II, as summarized in **Table 1**. PDT uses non-toxic photosensitizers and visible light at a specific wavelength in combination with oxygen to produce reactive oxygen species (ROS) that kill malignant cells. As a type II ICD inducer, it differs from type I in the CRT exposure pathway. After PDT, ATP release depends on ER stress and CRT exposure depends on ROS formation, but not on eIF2α (the eukaryotic initiation factor 2 α) phosphorylation, caspase-8 activation or ERp57 translocation which are the key features of type I ICD inducers [23].

**Table 1.** Classical ICD inducers.

	**Inducer**	**Cell line**	**Signals**	**Action**
**Typ I**	Bleomycin	MCA205, B16F10, CT26	ROS, DAMPs, ATP, ER Stress	ICD-induced IFNγ-dependent CD8 T cell response [106]
	Cyclophosphamide	BBL-5, B16	CRT, HMGB1	Immunogenic apoptosis, increased phagocytosis [107]
	Anthracyclines	CT26, B16F10, gp100 Mell, DU145, OV90, AML cells	CRT, HMGB1, ATP, HSP	ICD with autophagy, increase phagocytosis, DC activation, antitumor specific response [20, 26, 27]
	Oxaliplatin	CT26, RKO, HCT116	CRT, HMGB1, caspase-8	ICD induced, positive correlation with efficacy in colon cancer patients [108]
	Ionizing radiation	CT26, MCA205	CRT, HSP70	Partially dependent antitumor T cell response [109, 110]
	Cardiac glycosides	U2OS	CRT, ATP, HMB1, HSP90	ICD induction, increase survival in breast, colorectal, head and neck and hepatocellular carcinomas [111]
**Type II**	Oncolytic Virus	GL261 glioma cells	ER Stress, HMGB1, ATP, CRT	NK cell activation, macrophage and granulocyte infiltration [112]
	Photodynamic Therapy (PDT)	LLC, EMT6, C127, U87	ROS, Autophagy, HMGB1, HSPs, CRT	DC activation, adaptive immunity-dependent antitumor response [23]

Moreover, recent evidence places ER stress at the core of all the scenarios where ICD occurs, irrespective of the types. ER stress and unfolded protein response (UPR) are required for the CRT exposure on the cell surface during ICD and consist in the simultaneous activation of three pathways: i) the PERK (eukaryotic translation initiation factor 2 alpha kinase 3)-mediated phosphorylation of eIF2α, that induces the expression of ATF4 (activating transcription factor 4), ii) the translocation of ATF6 (activating transcription factor 6) from the ER to the Golgi and then to the nucleus, and iii) the IRE1α (endoplasmic reticulin to nucleus signaling 1)-mediated splicing of XBP1 (X-box binding protein 1). These three transcription factors activate a gene expression profile to facilitate apoptosis [29]. Thus, ER stress and the UPR have emerged as important targets in different human cancers, for example in multiple myeloma. In this hematological malignancy of mature antibody secreting plasma cells, the constant and elevated production of immunoglobulins leaves these cells heavily reliant on the activation of the UPR [30]. For this reason, drugs that disrupt ER homeostasis and engage ER stress-associated cell death, such as proteasome inhibitors as well as novel ER stressors, are intended to be promising therapeutic agents in this setting [30].

During tumor development, cancer cells have to cope with harsh conditions that trigger ER stress. Thus, UPR activation constitutes an important hallmark of several human cancers since it endows cancer cells with the ability to acquire essential characteristics required for tumor progression [31]. In transformed cells the need for protein and lipid production is increased for rapid proliferation for the adaptation to a particular oxygen and nutrient deprived environment. To overcome such challenges cancer cells, exploit intrinsic mechanisms such as UPR for survival. UPR signaling supports tumor development in several ways: i) by promoting cell cycle progression and cell proliferation pathways, ii) by promoting the expression of pro-angiogenic factors in response to hypoxia, iii) by inducing the recruitment of several types of cells into the tumor microenvironment to evade the immune response and iiii) by favoring the epithelial to mesenchymal transition by overcoming the stress of cell detachment [32].

### Natural products as non-classical ICD inducers

Two categories of natural products can be distinguished and considered as immune system activators: single compounds derived from natural sources or natural complex fractions composed of a large number of molecules. A non-exhaustive overview of these products is presented in **Table 2**.

**TABLE 2.** Natural products reported as ICD inducers.

	**Inducer**	**Cell lines**	**Signals**	**Action**
**Single molecules**	Hypericin (*Hyperian perfotum*) combined with PDT	T24, CT26, GL261	ER Stress	ICD induced, tumor growth inhibition [39]
	Shikonin (*Lithospermum erythrorhizon*)	B16	DAPMs, tumor antigens	ICD induced, DC activation, Th1/Th17 specific response [37]
	Wogonine (*Scutellaria baicalensis*)	MFC	CRT, ATP, HMGB1, ROS, ER stress	ICD induction, increase lymphocyte counts, tumor growth inhibition [113]
**Complex formulations**	P2Et (*Caesalpinia spinosa*)	B16F10, 4T1	CRT, ATP, HMGB1, autophagy, ROS, caspase 8, mitochondrial membrane depolarization	ICD induction, multifunctional T cell response and chemotherapy adjuvant (4T1). Specific T CD8 response (B16). [42, 43, 102]
	*Hemidesmus indicus* decoction	DLD	ER stress, ATP, HMGB1, CD83	DC maturation after ICD induction [40]

#### Single molecules

Shikonin (SK), a secondary plant metabolite isolated from *Lithospermum erythrorhizon*, has been described as having several biological activities that include the inhibition of the promoters of pro-inflammatory cytokines such as TNF-α [33] and GM-CSF [34] and the splicing of TNF-α pre-mRNA [35]. Moreover, for SK and its derivatives antitumoral properties through ICD induction have been described [36]. In fact, SK has been shown to induce the expression of different DAMPs including CRT, HMGB1, GRPp78, HSP70 and HSP90 in several tumor cell lines. Moreover, tumor cells treated with SK and lipopolysaccharide (LPS) activate DCs and induce the differentiation of tumor-specific Th1 and Th17 CD4 T cells [36]. Further studies showed that the heterogeneous nuclear ribonucleoprotein A1 (hnRNPA1) is also a specific protein target of SK and that its suppression is necessary for the expression of the signals that play a major role in ICD induction. All together these reports demonstrate the multi-target capacity of SK [37]. A second example of an isolated compound is Hypericin, an anthraquinone derivative which in combination with Hyperforin is one of the principal components of the *Hypericum* flower plant, commonly named Saint John's wort. Recently, the use of DC vaccines in combination with hypericin-based PDT (Hyp-PDT) was shown to induce ICD and was tested to treat Hight Grade Gliomas (HGG) in an animal model. It could be shown that the DC vaccine:Hyp-PDT combination improved survival of tumor-bearing animals, and that the effect was dependent on cell-associated ROS production and the release of DAMPs [38], including extra-cellular HMGB1 and cell surface CRT [39]. These findings suggest that Hyp-PDT–based anticancer vaccines are worth additional assessments for clinical use in the future.

#### Complex fractions

Complex fractions can have immunomodulatory capacities and antitumor activity as indicated recently by Turrini *et al.*, who showed that a decoction from plant roots of *Hemidesmus indicus* is able to induce ER stress along with the hallmarks of ICD to stimulate DC maturation and the upregulation of CD83 as a costimulatory signal [40]. Moreover, P2Et, a gallotannin-rich fraction extracted from the red fruits of *Caesalpinia spinosa,* was initially reported for its cytotoxic properties on several cancer cell lines [41]. P2Et has been previously characterized as a complex polyphenol-rich fraction with antitumoral activity and was shown to contain a large amount of gallic acid after acid hydrolysis, mainly due to the presence of gallic acid derivatives like galloyl quinic acids. Chromatographic fingerprint using HPLC/PDA revealed that most compounds in the fraction absorb at a wavelength of 254 nm, which is characteristic for some polyphenolic compounds and gallotannins [42]. More recently, complementary studies have shown that P2Et induces immunogenic apoptosis through the expression of extracellular CRT, HMGB1, ATP and autophagy in preclinical models of breast carcinoma [42] and metastatic melanoma [43]. Furthermore, P2Et not only induces the expression of the cellular markers of ICD but also activates an immune-dependent antitumor activity. As for SK, P2Et treated cancer cells act as a therapeutic vaccine that delays tumor growth in the B16-F10 melanoma model, through the induction of functional antigen-specific CD8 T cells [43]. In addition, it was also recently shown that the ICD induced by P2Et is due to ER stress and PERK-dependent calcium release [44].

## NATURAL PRODUCTS AS ICD-INDEPENDENT ACTIVATORS OF THE IMMUNE SYSTEM

Plant- or animal-derived natural products have different biological properties that can induce or sustain immune responses while also targeting tumor cells through ICD-independent mechanisms. For example, natural single molecule derivates such as phyllanthusmin C, curcumin, chitosan and methyl gallate derivatives possess immunoregulatory properties influencing the innate and/or adaptive antitumor immunity. The Ellagic acid peracetate derivate from methyl gallate found in wine (among other sources), is able to significantly reduce tumor growth in the B16-F10 melanoma mouse model by increasing the number of immune cells, while inducing tumor cell death via the decrease of the anti-apoptotic BCL-2 protein [45]. Further, chitosan present in high quantities in shrimp shells has been reported as a non-toxic natural compound with cytotoxic capacity against the sarcoma 180 (S180) tumor cell line and as an innate immune system activator [46]. The treatment with chitosan in leukemia and B16-F10 models showed direct activity on DCs by inducing IL-12 and IL-15 secretion followed by STAT4 and NFkB activation in natural killer (NK) cells to increase their survival, cytotoxicity and IFN-γ production [46]. Conversely, Phyllanthusmin C isolated from *Phyllanthus reticulatus* acts directly on NK cells by promoting NFκB signaling enhancement and finally inducing IFN-γ secretion regardless of the DC activation or the presence of IL-12 or IL-15 in the media [47].

The activation of the adaptive immune system can be induced by both single compounds, as curcumin, and natural complex fractions, as herbal medicine juzen-taiho-to (JTT). On one side, Curcumin derived from *Curcuma longa* has been reported as having antitumor activity through the induction of apoptosis in tumor cells [48] but also, and very importantly, by attenuating the immunosuppressive tumor microenvironment *in vivo*. In 2012 Chang *et al.* demonstrated that the *in vivo* treatment with curcumin in combination with T cell transfer increases tumor infiltration and restores the antitumor activity of transferred CD8 T cells by inhibiting IDO and TGFβ production by tumor cells [49]. On the other side, a complex mixture composed of ten different herbs called JTT, which is widely used in clinics in Japan, has been shown to activate the adaptive immune response *in vivo* in tumor models. Using the RET-transgenic melanoma mouse model, in 2001 Dai *et al.* showed that oral administration of JTT significantly delayed tumor growth and improved survival. They proposed a model where JTT does not act directly on the tumor cells but rather through T cell cytotoxicity [50].

## EFFICACY LOSS BY REDUCING THE COMPONENTS OF COMPLEX FRACTIONS

Guided fractionation is commonly used to study complex botanical preparations and determine their biologically active compounds. *Petiveria alliacea Linn* (*P. alliacea*) is commonly used in traditional medicine in Central and South America and the Caribbean cost. Infusions made with the leaves and stems of this plant have been used to treat leukemia and breast cancer by the native communities [51]. However, the mechanism of action in cancer has been only partially described. The work of ***Hernandez*** *et al.* showed that the plant extract obtained from the leaves of *P. alliacea* induces breast cancer cell death through the modulation of their glycolytic metabolism 52, 53]. This extract was characterized by HPLC and the principal components were identified. Several subfractions from the original one (guided fractionation) were evaluated for their antitumor activity and the IC50 revealed lower biological activity compared to the original fraction. These findings support the importance of the complex interaction among components (*Susana Fiorentino, personal communication*).

Another significant indication of this phenomenon is illustrated by the work by Junio *et al.* were they evaluated the properties and the components of a botanical medicine made from *Hydrastis canadensis* (Goldenseal) [54]. This preparation is ranked among the 20 most commonly used herbal preparations worldwide and is recommended to treat viral and bacterial infections and diarrhea. Goldenseal extracts from leaves and roots are rich in alkaloids like berberine that is reported to have antimicrobial activity. However, previous work by the same group showed that berberine alone has only little biological activity and that in the mixture, other compounds might play a role [55]. Further investigations showed that the extract was also rich in flavonoids which could block multidrug resistance efflux pumps and thereby increase efficacy in the absence of antibacterial properties. Their work described the directed fractionation of the original preparation to characterize the components, demonstrating that single compounds do not fully account for the biological activity of the main preparations 55].

As illustrated in this section, natural products represent a versatile “chemical toolbox” of potent medicines with verified health promoting active principles (**Table 2** and **Table 3**). However, a consistent issue in research on natural products is the lack of clarity surrounding their mechanism(s) of actions and/or their exact composition [56]. Since each natural product is capable of binding to multiple proteins with unrelated structures, each small molecule may have multiple targets. In that respect, an interesting question remains: can we profit from this multi-target property or should we avoid it?

**TABLE 3.** Other immune system activating natural products.

	**Inducer**	**Model**	**Signals**	**Action**
**Single molecules**	Phyllanthusmin C	Human primary NK cells	IFNγ	NK activation [47]
	Curcumin	E.G7	Reduction of IDO and TGFβ	Increase of CD8 T cell antitumor effector functions [49]
	Chitosan	Sarcoma 180, B16F10	IL-12, IL-15, IFNγ	DC and NK activation [46]
	Methyl Gallate derivates	B16F10	Reduction of BCL2 proteins	Apoptosis induction and increased immune cell numbers [45]
**Complex formulations**	Juzen-taiho-to	Mel-Ret		T cell activation and enhanced cytotoxic activity [50]
	*Astragalus membranaceus*	Spleen cells	TNF, IL-6	Spleen cell proliferation, B cell response, Alloantigen specific CTL response, Macrophage activation [114]

## MULTI-TARGET PHYTOTHERAPY IN CANCER: PROS AND CONS

As mentioned above, one of the main characteristics of botanical extracts relies on their complexity, rendering the identification of all molecular targets within the extracts difficult to be determined. But how do these complex preparations work? It might be speculated that they are able to modify different proteins to a certain extent within the same network, but it is also possible that weak effects induced by the whole are sufficient to have a pharmacological effect without having identified a major compound. Almost 20 years ago few pioneering groups reported the possible benefit of the multitarget profile of biological active small molecules [57, 58]. Since then, the field of polypharmacology and multi-target drug discovery have been growing, and have proposed new concepts to overcome the major limitations of classical “one target, one drug” strategies [59]. Instead of single target agents, the identification of molecules or combinations that have multiple interconnected targets or act on similar networks might be beneficial.

Herbal formulations are mainly composed of multiple biologically active molecules and several studies have proven their benefits in different types of diseases. However, they are not fully accepted as standard treatments mainly due to their complex nature and the lack of stringent quality control in their preparation [60].

The primary aim of traditional phytotherapy is to use plant extracts to create complex combinations, where even the “single” extracts are mixtures of different molecules. As a result, in China, formulations of complex botanical products have been evolving overtime, with more than 100,000 currently being documented. Instead, western pharmacologists are mostly confused by their complexity, although several combination therapies are commonly used for the treatment of pain [61], AIDS [62, 63] or cancer to prevent chemoresistance [64], highlighting the advantages of multi-target approaches.

One of the best examples to better illustrate the potential multi-target activity of natural products comes from the analysis of the multiple bioactivities of green tea preparations. To date, a significant number of studies has demonstrated that green tea has health beneficial effects which include reduction of serum cholesterol, prevention of low-density lipoprotein oxidation and decreased risk of cardiovascular disorders and cancer [65]. Many of these effects are most likely mediated by polyphenols contained in green tea, such as flavonoids and flavonols. One of the most studied flavonols is the epigallocatechin-3-gallate (EGCG) that has been reported for its anticancer activity and the potential to interfere with multiple signaling pathways in cancer cells [66]: i) by binding to the BH3 pocket of Bcl-2 family proteins to inhibit their antiapoptotic function [67]; ii) by inhibiting the NFκB activity and MAPK pathway in human colon cancer cells [66]; iii) by the specific inhibition of the chymotrypsin-like activity in the proteasome *in vitro* and *in vivo* [68]; iv) by the inhibition of the matrix metalloproteinases (MMP) 2 and 9 in the prostate of TRAMP mice [69].

As for the P2Et example, this fraction includes in its composition gallotannins-like ethyl gallate and gallic acid in high proportion. The comparison of the effect of the full fraction to the isolated compounds showed that the single molecules have very different effects on autophagy, being individually less effective in inducing the DAMPs required for ICD in the cancer therapy context. To obtain results comparable to the whole extract, almost 20 times more of the single molecules is required, which in turn might result in increased toxicity (*Alfonso Barreto, personal communication*). Hence, attempts to reproduce the effects of complex formulations by single molecules may prove unsuccessful.

However, as in the case of drug combinations, molecular interactions in complex plant formulations might be antagonistic or dangerous since botanical preparations can contain toxic or even lethal compounds. Some compounds such as Pyrrolizidine alkaloids, secondary metabolites, need to be removed due to their hepatotoxicity [70] or aristolochic acid is known to promote kidney nephropathy [71]. Moreover, the increased use of herbs for self-medication is resulting in opposite outcomes depending on the context [72], as recently reported for the P2Et fraction. As mentioned above, this fraction was initially described to induce ICD and immune system activation in preclinical tumor mouse models [42, 43] but additional studies showed pro-tumoral effects if used in a prophylactic context [73]. These examples reinforce the need to be proactive to develop appropriate measures to test the safety, the quality and the mechanisms of action of public accessible natural products.

## NETWORK PHARMACOLOGY: MULTI-TARGETING AND MULTI-DISEASE CONCEPT

To fight complex diseases such as cancer, cardiovascular diseases, AIDS or neurodegenerative disorders, the approach of single target drugs has shown discouraging results [74]. In contrast, multiple target drugs or combinations are more efficient and, in many cases, shows less toxicity [75]. The concept of signaling network analysis, or network pharmacology (NP), proposed initially by Hopkins in 2008, appears as a suitable tool to assess complex interactions and find new targets for “rational drug discovery”, by evaluating drug mechanisms of action through a biological network and its comparison with the target model [76]. Today, drug discovery could benefit from network-based approaches to overcome problems such as the lack of efficacy, the potential resistance to compounds with a single target and toxic interactions among different components [76, 77]. This new concept intends to improve the prediction of drug targets in public databases and promote the use of network models using high-throughput screening and bioinformatics.

NP analysis of traditional medicines is becoming a popular approach to identify treatments of complex and multifactorial diseases, as reported in several studies that applied NP to assess natural complex fractions composed of different ingredients, unknown targets and different pharmacological mechanisms [78–80].

While drug actions have been originally considered as a “lock and key system [81], a growing evidence is revealing a more complex picture. Yildirim *et al.* in 2007 described that there are many keys for one lock and that this phenomenon is quite common as proteins rarely function as isolated entities, whereas they rather act as part of a highly interconnected cellular network [82]. Re-thinking drug action in the context of network biology may provide insights into how we can improve drug discovery for complex diseases. Further, NP analysis predicts that modulating multiple proteins might be required to fight against complex and strong phenotypes in a more effective way that only deleting individual nodes, having only little effect on the whole disease network [2]. For example, the combinatorial therapies used against hepatitis C virus (HCV) were conceived on the hypothesis that targeting two different nodes of HCV replication may exhibit superior antiviral activity and reduce resistance compared to single drugs [81]; the recent increase of multi-target drugs to treat schizophrenia and major depressive disorders aims at targeting the complexity and multifactorial nature of such disorders [81, 83].

In the case of Traditional Chinese Medicines (TCM) the molecular mechanisms of action for many formulations remain unclear. To solve this problem a TCMAnalyzer was recently developed. This tool designed by Liu *et al.* can be used to identify the potential compounds responsible for the biological activity, to investigate the molecular mechanisms at a systematic level and to explore the potential targeted bioactive herbs [84].

The molecular network analysis of the TCM is not restricted to the identification of the mechanism of action; it has also been important to explain and study the compatibility or possible toxic interactions among the different compounds of complex preparations [77, 85]. In 2010 Li *et al.* proposed a method named the Distance-based Mutual Information Model (DMIM) to identify useful relationships among the herbal components of several complex formulas. DMIM combines mutual information entropy and “distances” between the different herbs to construct an herb network that allows to illustrate the traditional herbal pairing and compatibility [86]. Equally important, the concept of network toxicology uses the principles of NP for the understanding of the “toxic-gene-target-drug” interaction network to predict and estimate the toxicity and side effects of drugs or complex formulations [85]. Technologies that feature high-throughput screening, toxic compounds exclusion and drug-target network might be very important for active ingredient screening and toxicity assessment [85]. In the work of Fan *et al.*, NP was used to reconstruct the network of “compound-protein/gene-toxicity” to identify the toxic molecules and predict the side effects of the known compounds. This helps to improve the safety of natural complex preparations and to understand toxic mechanisms. Currently several databases for the study of network toxicology include CTD [87], TOXNET [88] and the National Toxicology Program [85], among others.

### Network pharmacology in cancer

Cancer is a multifactorial disease [89]. For this reason, the seek for multi-target drugs represents a promising approach to understand how signaling networks are altered in transformed cells and to identify pertinent targets during drug discovery [90]. For example, the NP approach was instrumental to identify altered autophagy-associated genes and to reveal which alterations in this pathway correlated with patient survival. Overall, this strategy might contribute to identifying the specific signaling pathways that drive pro- or antitumor signals [76]. For that purpose, Wong *et al.* used a computer-aided drug design (CADD) method applied simultaneously to multiple drug targets and systems biology [91]. They evaluated the core network markers of four cancer types (colon, liver, lung and bladder) by systems biology followed by computational analysis and identified 28 cancer housekeeping proteins. Using this information, wet-bench researchers combined the top ligands for each of the 28 core proteins and found that they could decrease the IC50 with concomitant increase in cell death using a multidrug approach with different targets [91]. Further, in the ICD context where ER stress induction with PERK activation are indispensable steps to initiate the immunogenic apoptotic axis [92] NP would allow to dissect the biological networks targeted by different therapeutic options (e.g. single molecules, complex formulations or combinatorial therapies). This would enable us to predict specific candidates capable of triggering ROS production, Ca^2+^ alterations or defaults in glucose consumption and contribute to propose *bona fide* ICD inducers with the characteristics mentioned in **Table 1**.

Another attractive possibility would imply the use of the network analysis to identify and prioritize several hubs associated with the response to immunotherapy, such as immune checkpoint blockade. In the previously cited work of Lesterhuis *et al.*, they identified context dependent gene expressions that were only visible after NP analysis. They also applied a drug repurposing approach to the response-associated network data and identified pleiotropic drugs that improved the response rate to anti-CTLA4 and that had never been associated with antitumor properties and could have never been predicted to work [93].

The complex tumor microenvironment and the immunomodulation that characterize it are often difficult to be addressed at the same time with a single therapeutic agent and several approaches remain unsuccessful or insufficient. For instance, autophagy's role and regulation during cancer development has been the topic of long debates due to the opposite effects reported in the literature. In line with this, the use of metformin to induce autophagy as effective cancer treatment has encountered difficulties during clinical trials [94]. Further, ER stress and UPR are involved in several types of cancer and they are frequently correlated with advanced stage and chemoresistance. UPR is important for changes in gene expression that can contribute either to prevention or survival during tumor development and can be modulated by chemotherapy but the clinical implications are only beginning to be understood [95]. Lastly, tumor infiltrating T cell impairment and hijacking of immune system effectors remain one of the most complex problems for immunotherapeutic approaches [96] despite the FDA approval of several immune checkpoint inhibitors [97].

In a similar way, natural products and NP approaches are being used to discover new agents or indicate new combinations to improve current therapies [98, 99]. Multitarget natural dietary supplements containing some ingredients such as Ginseng extracts, Grape seed extracts, and curcumin have been shown to promote recovery from severe illnesses, and relieve standard therapy-induced side effects [100]. Interestingly, clinical trials have also proven that some natural products can reduce chemotherapy and radiotherapy-induced toxicity [101]. Common gastrointestinal toxicity side effects of chemotherapy can be reduced by natural products. Red Ginseng decreases nausea, vomiting, dyspnea and fatigue after taxan and platinum-based chemotherapy [101].

Natural fractions not only help to counteract drug side effects. Their activity can also exert synergistic effects with conventional treatments and antitumor activity by overcoming drug resistance. In 2016 Sandoval *et al.*, showed that the P2Et fraction exerts synergistic activity with doxorubicin *in vitro* in several cancer lines and *in vivo* in a mouse model transplanted with a doxorubicin-resistant breast cancer cell line (TSA). Interestingly, P2Et extract revealed a synergistic effect with doxorubicin in resistant cell lines, and its effect does not seem to be mediated by ROS increase [102].

Another example is the combination of PHY906, a Chinese traditional complex formulation consisting of four different herbs, with Irinotecan (CPT-11) as treatment for metastatic colon or rectal carcinoma [103]. In 2010, Lam *et al.* used the MC38 mouse model to show that PHY906 increased the antitumor activity of CPT-11, while decreasing poor prognostic factors as weight loss and promoting intestinal recovery from the chemotherapy damage. PHY906 was promoting the expression of intestinal progenitor or stem cell markers through the Wnt/β-catenin pathway and at the same time inhibiting inflammation [60]. These results suggest that the development of complex, multi-component herbal medicines that are able to target multiple sites and activating different signaling pathways could be useful for future drug development and as an add-on for improving current standard treatments.

## CONCLUSIONS

Historically, natural products have been the source of a great variety of medicinal preparations and today they continue to provide prototypes for pharmacologically active compounds, particularly as anticancer and antimicrobial agents. According to the World Health Organization (WHO), 80% of people still rely on traditional plant-based medicines for primary health care and the plant-derived drugs remain related to their original ethno pharmacological usage. In addition, in this review we intended to stress the capacity of natural products to influence the immune system by either affecting the functions of immune cells or by directly acting on malignant transformed cells to turn them into intrinsic immune cell activators, showing the potential of natural products as immunomodulators. The results discussed here put in evidence the importance of dissecting the molecular mechanisms of the immunomodulatory effects of the natural compounds since this could lead to the discovery of novel and promising candidates that can be used in future immunotherapeutic strategies.

However, the complex composition and the multitarget capacities of natural products remains one of the main concerns for the western medicine due to safety and quality control reasons. Nevertheless, NP approaches as the ones mentioned here could help to put in place standardization protocols of proved bioactive compounds or formulations to elucidate the molecular mechanisms of action and therefore facilitate their inclusion in clinical trials and their commercialization.

In order to promote and improve the quality of herbal medicines and to reduce the proportion of adverse events attributable to their poor quality, the WHO has also committed to the development of a series of technical guidelines related to quality assurance and control of herbal medicines [104]. Finally, the European framework has provided a powerful regulation model for the harmonization of scientific assessment and facilitation of product marketing [105].

## Abbreviations:

CRT: – calreticulin;
DAMP: – damage associated molecular pattern;
DC: – dendritic cell;
DMIM: – Distance-based Mutual Information Model;
ER: – endoplasmic reticulum;
HCV: – hepatitis C virus;
ICD: – immunogenic cell death;
JTT: – juzen-taiho-to;
NK: – natural killer;
NP: – network pharmacology;
PDT: – photodynamic therapy;
ROS: – reactive oxygen species;
SK: – shikonin;
TCM: – Traditional Chinese Medicine;
UPR: – unfolded protein response.
